# A case report of giant retroperitoneal liposarcoma

**DOI:** 10.1097/MD.0000000000041923

**Published:** 2025-03-21

**Authors:** Zicheng Bao, Nan Jia, Zhidong Zhang, Pingan Ding, Qun Zhao, Xuefeng Zhao, Yong Li

**Affiliations:** a Third Department of Surgery, Fourth Hospital of Hebei Medical University, Shijiazhuang 050011, China.

**Keywords:** giant size, retroperitoneal liposarcoma, soft tissue tumor, surgical approach

## Abstract

**Rationale:**

Retroperitoneal liposarcoma (RPLS) is a rare malignancy with a high recurrence rate. Giant RPLS (≥30 cm) poses significant surgical challenges due to its invasive nature and compression of vital organs. Early diagnosis and multidisciplinary management are critical yet underreported in young patients. This case highlights the complexity of managing a 36-year-old male with a 60 cm × 50 cm × 40 cm tumor, emphasizing the importance of surgical precision and collaborative care.

**Patient concerns:**

The patient is a 36-year-old male who was admitted to the hospital due to abdominal distension presenting for over 7 months, accompanied by a rapid increase in abdominal girth for over the past 2 months. Based on his symptoms, physical examination findings, and imaging studies, the possibility of a giant retroperitoneal liposarcoma was considered.

**Diagnoses:**

Giant retroperitoneal liposarcoma.

**Interventions:**

The treatment involved the resection of the massive retroperitoneal tumor, along with partial resection of the descending colon, left ureterostomy drainage, and release of intestinal adhesions.

**Outcomes:**

The patient was discharged 14 days postoperatively with no immediate complications. Albumin levels improved to 35.2 g/L at 1-month follow-up. Telephone follow-ups at 3, 6, 12 months, and 5 years revealed no signs of recurrence on abdominal ultrasound.

**Lessons:**

The tumor had already invaded the surrounding organ tissues at the time of discovery in this case. Comprehensive preoperative evaluation, multidisciplinary collaboration in diagnosis and treatment, precise surgical techniques, and standardized intraoperative and postoperative management are essential to enhance surgical resection rates, minimize complications, and decrease the likelihood of postoperative recurrence.

## 1. Introduction

Liposarcoma (PLS) is a rare malignancy, accounting for 0.07% to 0.2% of all tumors, with approximately 12% to 40% occurring in the retroperitoneal space. Most patients are between 40 and 60 years of age.^[1]^ The primary clinical presentation of PLS is painless, progressively expanding mass. At the time of initial diagnosis, around 50% of patients have tumors larger than 20 cm in diameter. Liposarcomas with a diameter of ≥30 cm or a weight of >20 kg are typically classified as giant liposarcomas.^[2]^ Due to its insidious onset, PLS is often difficult to detect in its early stages. Patients typically seek medical attention only after the tumor has grown large enough to compress multiple organs, making surgical resection highly challenging. Enhanced computed tomography (CT) and magnetic resonance imaging (MRI) are valuable tools for evaluating the size of the tumor and its invasion of abdominal organs and blood vessels. Since PLS shows limited sensitivity to chemotherapy and radiotherapy, and no targeted drugs or immune therapies have been clearly established, surgery remains the primary treatment option. However, reducing the recurrence rate after surgery and minimizing the need for subsequent surgeries remain significant challenges that warrant further discussion.

## 2. Case report

**Figure 1.**
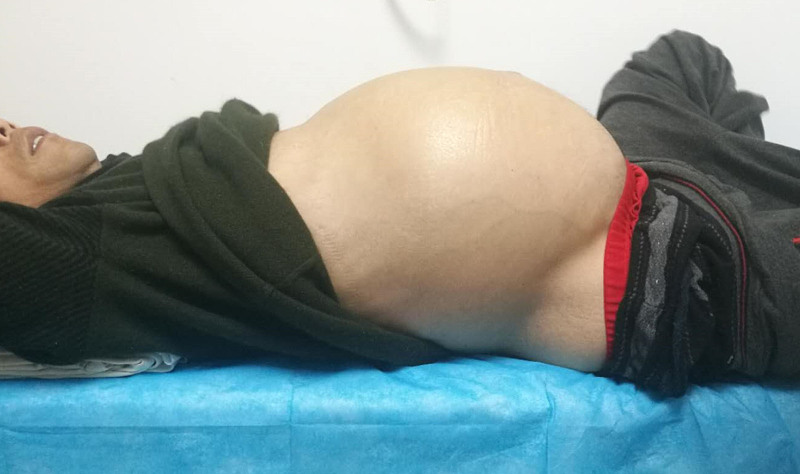
Preoperative abdominal appearance.

**Figure 2.**
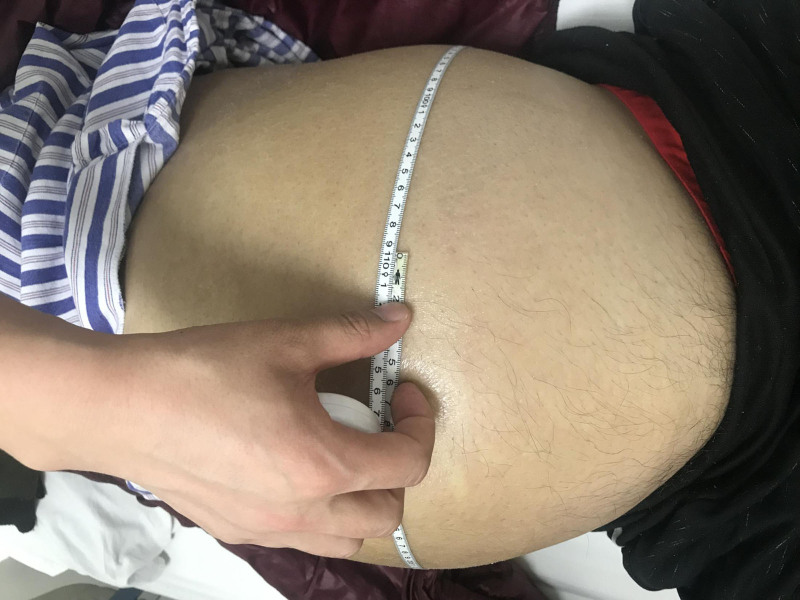
Preoperative abdominal circumstance.

The patient was a 36-year-old male who presented with abdominal distension lasting over 7 months, along with a rapid increase in abdominal girth over the past 2 months. He was admitted to Hebei Medical University Fourth Hospital in April 2019. He was 174 cm tall with a weight of 74.5 kg and a body mass index of 24.6, indicating emaciation. Physical examination revealed a diffusely distended abdomen, extending from the costal margin to the symphysis pubis, with a palpable mass of ill-defined borders but no tenderness (Fig. 1). The abdominal circumference measured 106.5 cm (Fig. 2), and there was noticeable edema in both lower limbs, with the left lower limb circumference of 36.5 cm and the right at 37.4 cm.

**Figure 3.**
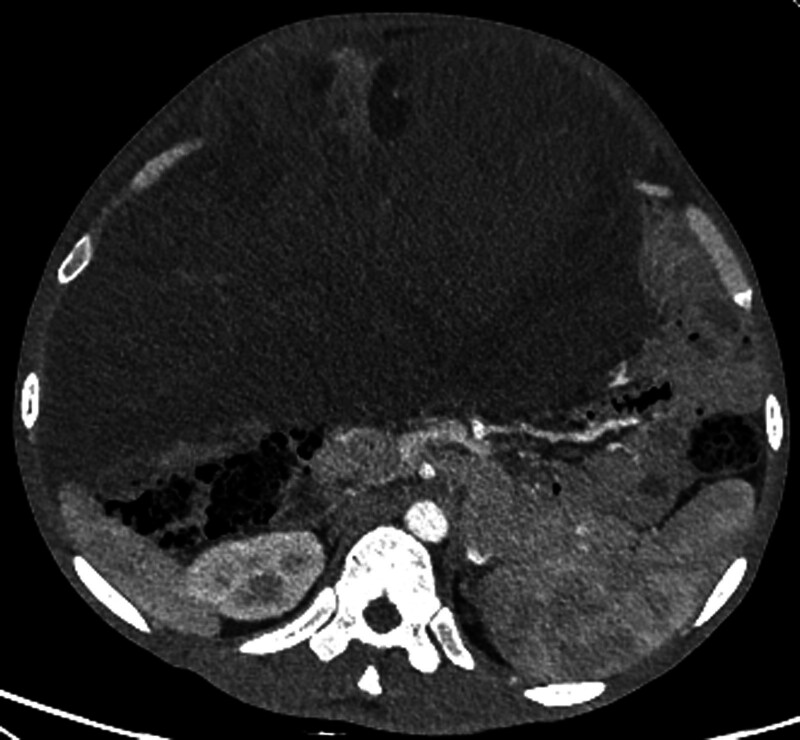
Contrast-enhanced CT. The CT scan reveals a heterogeneous mass in the retroperitoneum, with a maximum diameter of 35 cm, uneven density, and invasion of surrounding organs. CT = computer tomography.

**Figure 4.**
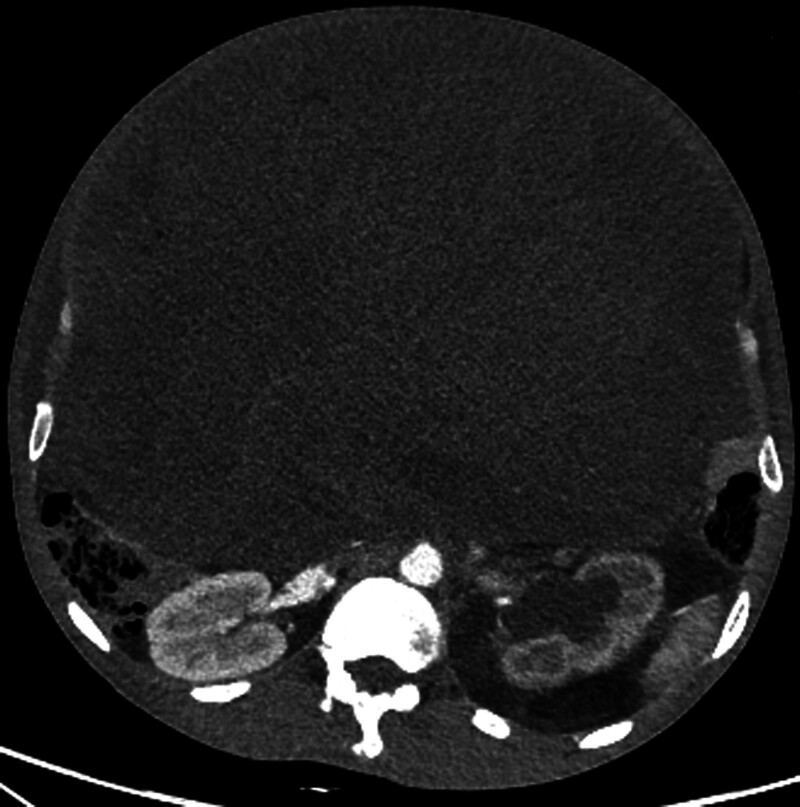
Contrast-enhanced CT. The CT scan reveals a heterogeneous mass in the retroperitoneum, with a maximum diameter of 35 cm, uneven density, and invasion of surrounding organs. CT = computer tomography.

An abdominal enhanced CT scan revealed an irregular soft tissue mass with uneven density, measuring approximately 35.2 cm along its long axis. The mass exhibited heterogeneous enhancement of the solid components on contrast-enhanced images (Fig. 3). Fat density in the pelvic cavity has increased, forming a mass-like structure that compressed the surrounding intestines (Fig. 3). The mass encircled the left kidney, causing dilatation and hydronephrosis of the left renal pelvis and upper ureter (Fig. 4). Blood vessels supplying the mass partly originated from a fine branch of the abdominal aorta, and the superior and inferior mesenteric arteries were seen within the mass margins. Renal function tests indicated decreased renal blood flow and mildly impaired filtration and excretion functions of the left kidney. The blood biochemistry report shows: albumin 22.8g/L; prealbumin 53.9mg/L. This indicates that the patient has hypoproteinemia and has been in poor nutritional status recently due to tumor compression symptoms.

A multidisciplinary team from general tissue surgery, urology, vascular surgery, and radiology convened to discuss and formulate the treatment plan. The vascular surgery team emphasized the need to carefully consider the anatomy of critical vessels, such as the inferior vena cava, and the relationship of the tumor to the surrounding intestines and ureter. The urology team highlighted the significant compression of the left ureter by the tumor. To minimize the risk of intraoperative ureteral injury, it was recommended to place bilateral double-J (DJ) ureteral stents preoperatively. After adequate preoperative preparation, the patient underwent surgery on April 26, 2019. The procedure involved a laparotomy to remove the large retroperitoneal tumor, combined with partial resection of the descending colon, left ureterostomy drainage, and release of intestinal adhesions under general anesthesia.

**Figure 5.**
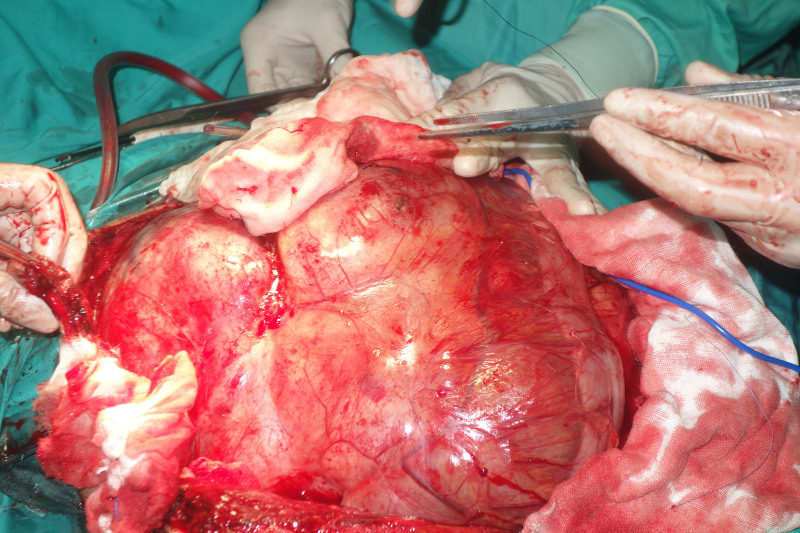
Abdominal exploration, the tumor occupies most of the abdominal cavity, with part of the descending colon being lifted up and tightly adherent to the surface of the tumor, exhibiting extreme tension. The mesocolon is relatively thin, and blood supply to some parts of the intestinal tube is poor.

**Figure 6.**
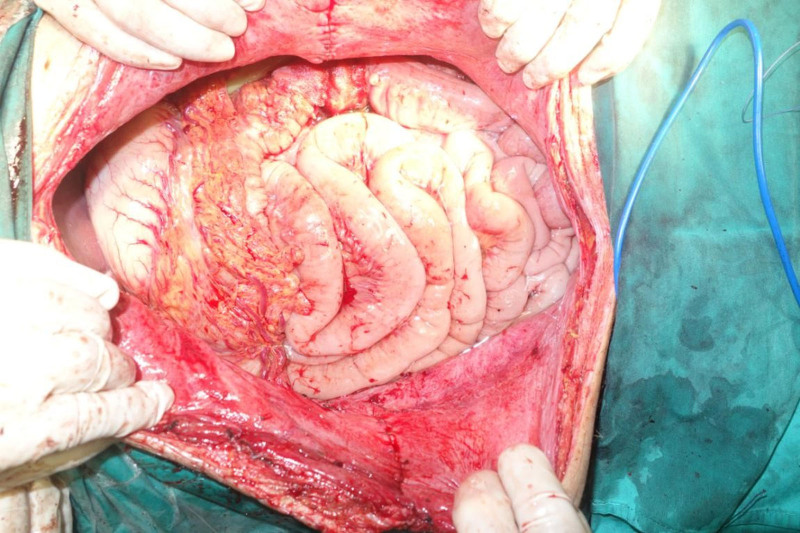
Restoration of abdominal organs.

**Figure 7.**
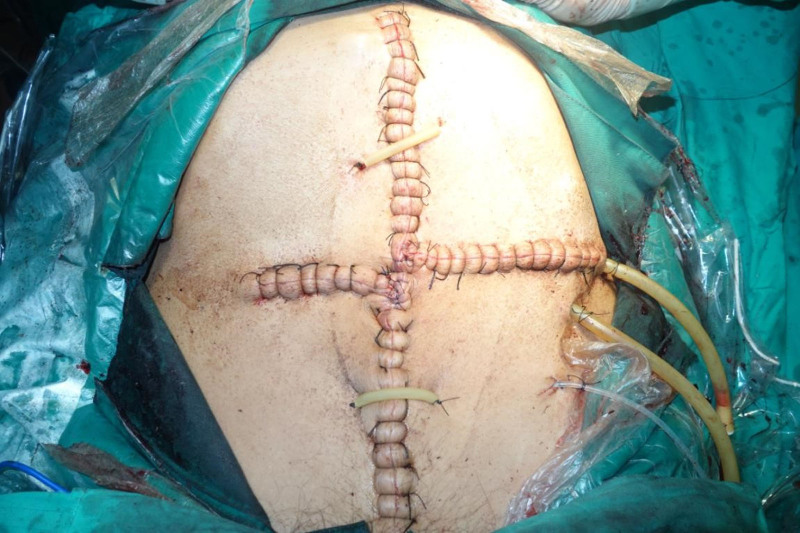
Abdominal incision and drainage tube.

Intraoperative exploration revealed a massive tumor occupying most of the abdominal cavity (Fig. 5). The descending colon was displaced, its walls edematous, and it was tightly adhered to the tumor with high tension. The mesocolon was thin, and certain intestinal segments had inadequate blood flow. The transverse colon and small intestine had been pushed into the right upper abdomen. The upper portion of the tumor compressed and tightly enveloped the left ureter. During the dissection, thermal damage occurred to the upper segment of the left ureter, necessitating a prophylactic ureterostomy. The abdominal cavity was then irrigated with warm distilled water, the abdominal organs were repositioned (Fig. 6), drainage tubes were placed, and the abdominal incision was closed with a “T-shaped” suture (Fig. 7).

**Figure 8.**
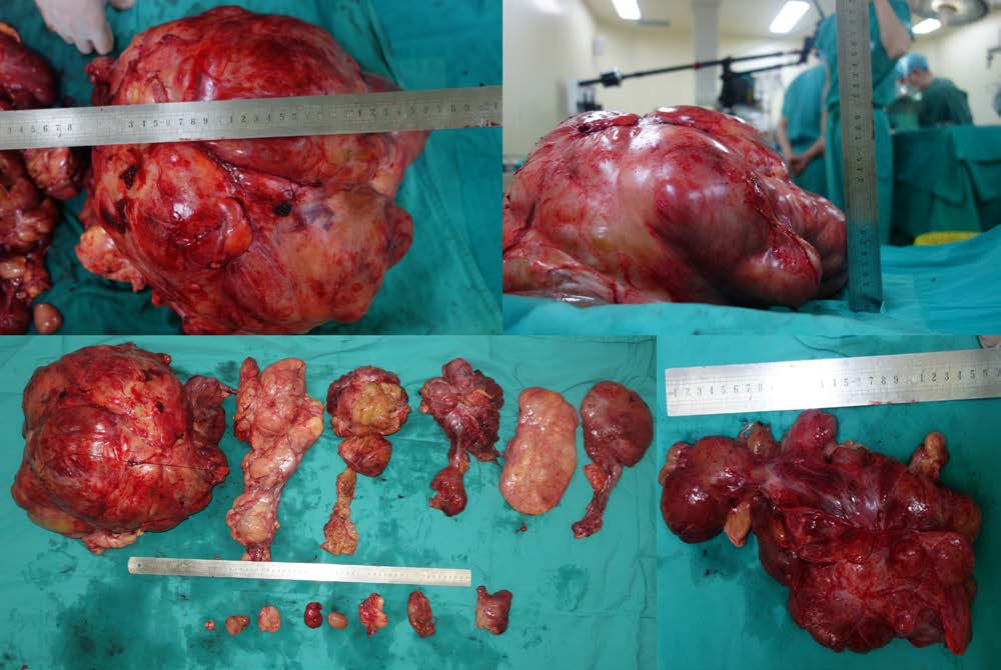
Gross photo of postoperative specimen.

**Figure 9.**
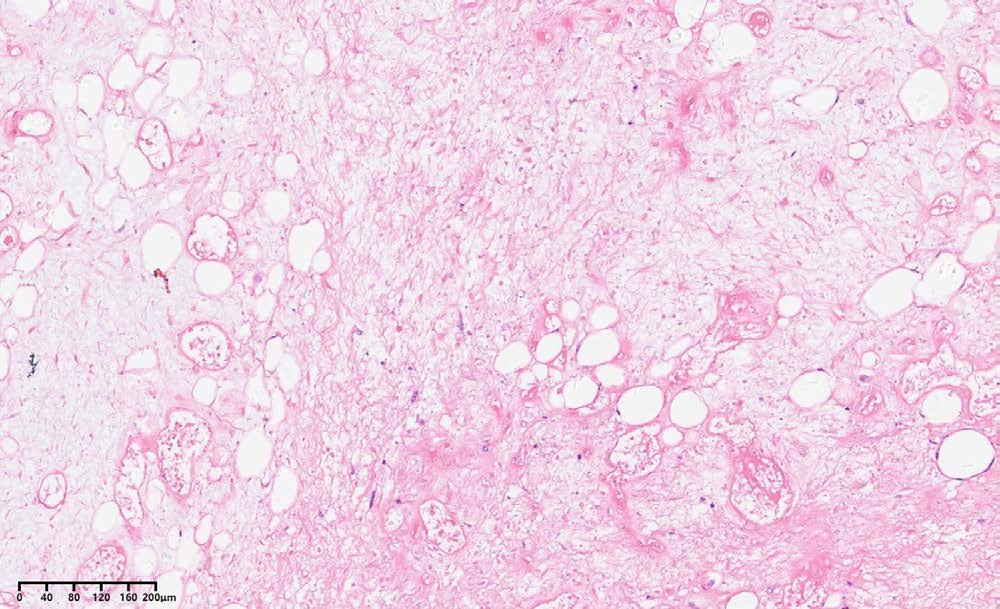
Hematoxylin and eosin staining × 100.

Postoperative pathology: The tumor measured 60 cm × 50 cm × 40 cm, with a total specimen weight of 20.5 kg (Fig. 8). The cross-section appeared tough and delicate, with a grayish-white color. Under a light microscope, highly differentiated liposarcoma components were observed transitioning into malignant nonfatty components arranged in bundles. The cells were spindle-shaped with mildly deformed nuclei (Fig. 9).

**Figure 10.**
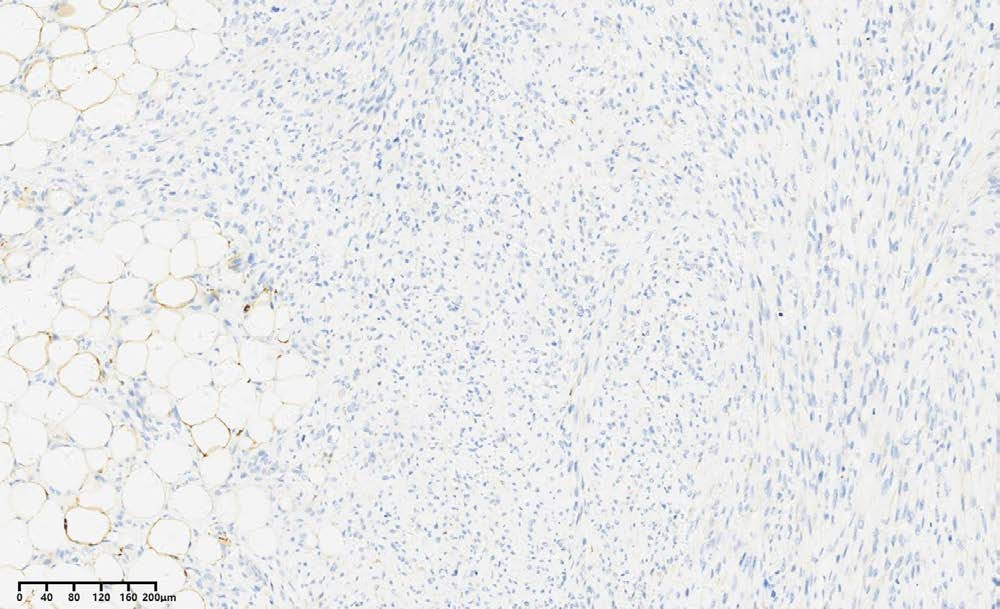
S-100 immunohistochemistry envision × 200.

**Figure 11.**
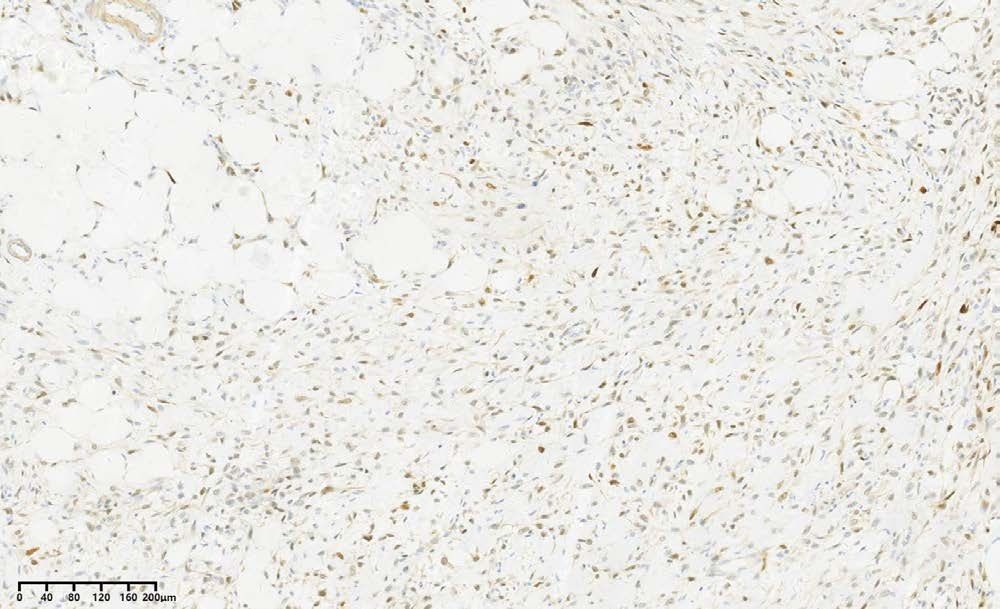
MDM2 immunohistochemistry envision × 200.

**Figure 12.**
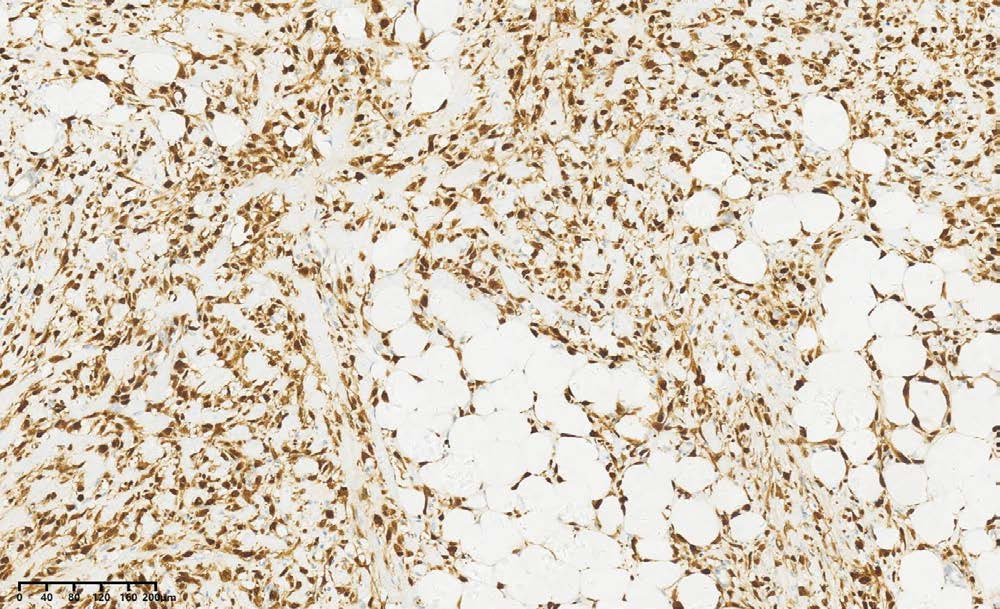
CDK4 immunohistochemistry envision × 200.

**Figure 13.**
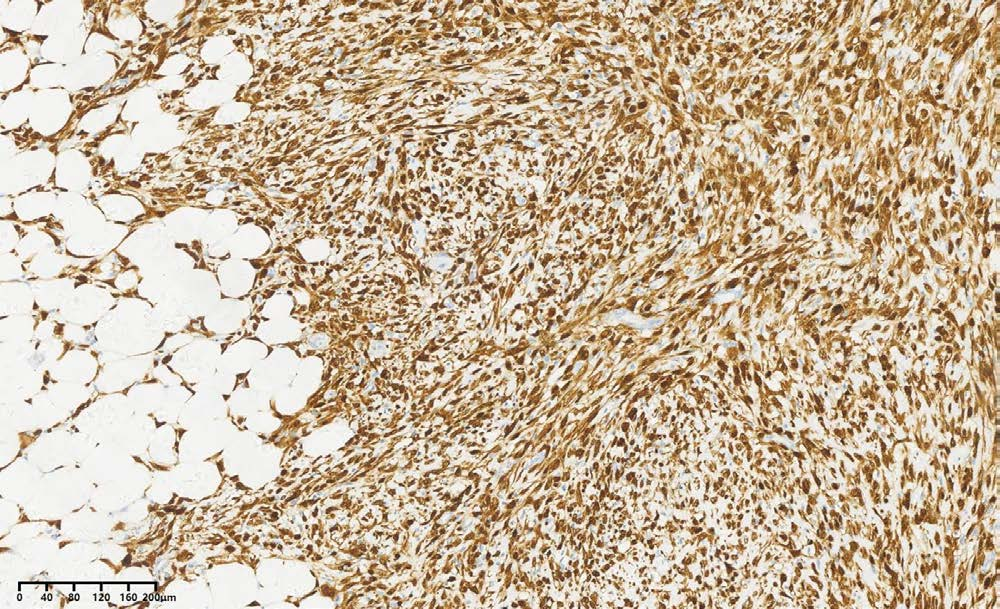
P16 immunohistochemistry envision × 200.

Immunohistochemical results were as follows: CD34 (blood vessel positive), S-100 (positive) (Fig. 10), MDM2 (positive) (Fig. 11), Des (partially positive), SMA (partially positive), Ki67 (40% positive cells), CD68 (negative), CDK4 (positive) (Fig. 12), P16 (positive) (Fig. 13). Based on the Deacu scoring system (2023), this case meets the diagnostic criteria for dedifferentiated liposarcoma (with a score of 6).^[3]^ No significant recurrence was detected during the postoperative telephone follow-up.

## 3. Discussion

Liposarcomas typically occur in the abdominal cavity, retroperitoneal space, and extremities where fat tissue is abundant. While they generally grow expansively, their growth rate is relatively slow compared to other malignant tumors. However, studies have shown that the growth rate of the same tumor varies during different periods.^[2]^ According to the World Health Organization Classification and Typing of Soft Tissue Oncology (2020 edition),^[4]^ adipose tissue tumors are categorized as benign, intermediate (locally aggressive), or malignant. Malignant liposarcomas are further divided into 4 subtypes: well-differentiated, dedifferentiated liposarcoma, myxoid/round cell liposarcoma, pleomorphic liposarcoma, and nonspecific liposarcoma. This case was diagnosed as dedifferentiated liposarcoma.

The onset of PLS is often insidious, and by the time the tumor is detected, it is typically large. In this case, the patient initially experienced no obvious symptoms, and the tumor measured nearly 20 cm at the time of diagnosis. Because the basic quality of life of the patient had not been significantly affected and the family faced financial constraints, both the patient and his family did not take the condition seriously, resulting in a missed opportunity for early treatment.

Early detection of RPLS requires a variety of imaging tests. Ultrasound is a noninvasive and cost-effective screening tool. However, CT is the preferred choice for auxiliary examination. A combination of CT plain scan, contrast-enhanced scan, and 3D reconstruction, as well as CT urography and CT angiography, can provide a detailed view of the tumor, including its effect on and displacement of abdominal and retroperitoneal blood vessels.^[5]^ MRI has limited coverage and spatial resolution compared to CT, making it less effective in visualizing fine structures.^[6]^ Studies have shown that CT and MRI exhibit different imaging characteristics depending on the pathological subtype of RPLS, but there is no significant difference in diagnostic accuracy between the 2 modalities.^[7]^

However, in cases where imaging suggests that the tumor is unresectable or when the tumor is suspected to be lymphoma, Ewin sarcoma, gastrointestinal stromal tumor, or another tumor type, a biopsy can provide critical information for treatment planning.^[8]^

Dedifferentiated liposarcoma needs to be differentiated from lipoma and atypical lipomatous tumor. In this case, the amplification of MDM2 and the degree of atypia support the diagnosis of liposarcoma, which is consistent with the criteria for dedifferentiated liposarcoma proposed by Deacu et al.

Surgical resection remains the most effective treatment for PLS, with complete tumor removal being crucial to reduce postoperative recurrence. Serio et al first introduced the concept of complete resection of macroscopic tumors, emphasizing that complete removal significantly impacts the survival of the patients.^[9]^ However, in a retrospective study, Linehan et al found no statistically significant difference in local recurrence rates between patients with negative surgical margins and those with positive margins.^[10]^ On the other hand, studies by Lewis et al^[11]^ and Bonvalot et al^[12]^ found that the presence of tumor cells at the surgical margins significantly affects local recurrence rates and the survival of patients. The 2016 expert consensus on the diagnosis and treatment of RPLS, published by the Professional Committee of Retroperitoneal and Pelvic Floor Diseases of the Chinese Society of Research Hospitals, recommended complete removal of the tumor within the scope of surgical adaptation, prioritizing safety. Given the anatomical complexity and histological characteristics of this tumor, achieving complete resection while adhering to safe surgical margin standards is essential. The scope of surgery largely depends on the experience and judgment of the surgeon. Recent surgical approaches advocate for expanding the extent of resection to ensure complete removal of the tumor.

Strauss et al^[13]^ reported that the 5-year local recurrence rate after complete surgical resection was 55%, with a survival rate of 75%. Additionally, postoperative major complications occurred in 18% of cases, early reoperation was required in 12%, and postoperative mortality was 3%.^[14]^ Bao et al suggested that patients with RPLS who exhibit leukemoid reactions tend to have a poor prognosis. Besides regular imaging, hematological examinations preoperatively can help monitor the prognosis in such patients.^[15]^ While palliative surgery to remove part of the tumor may not significantly improve the overall survival of patients, it can enhance the quality of life and potentially prolong survival for patients with well-differentiated liposarcoma or those with large tumors causing severe symptoms or life-threatening conditions. In such cases, reducing tumor load provides an opportunity for further antitumor therapies.

Liposarcoma is generally not responsive to traditional radiotherapy and chemotherapy,^[16]^ but with the advancements in medical technology, new clinical exploratory studies and treatment approaches are emerging. Research by Tuan et al^[17]^ and Ecker et al^[18]^ has shown that combining tumor resection with radiotherapy can improve local tumor control and increase the survival rates of patients undergoing surgery. Given the high recurrence rate of PLS, targeted therapy is an area of ongoing investigation. Currently, certain genes carried on chromosome 12 – such as MDM2, CDK4, SAS, and HMGA2 – are known to be associated with the development of PLS. In 2022, researchers found through whole-genome sequencing and transcriptome analysis that they had uncovered driver genes (such as MDM2 and CDK4 amplifications) and potential therapeutic targets for different subtypes of liposarcoma.^[19]^ Lina et al also investigated the role of MDM2 amplification in the diagnosis of DDLS and its impact on prognosis.^[20]^ And studies have shown that CDK4/6 inhibitors demonstrate survival benefits in advanced DDLS, but the challenge of patient resistance remains unresolved.^[21]^ Studies have shown that hairpin RNA, which directly targets these genes, can inhibit the proliferation of liposarcoma cells in vitro.^[22]^ Additionally, the introduction of targeted drugs like tribetidine for FUS-CHOP gene fusion in myxoid liposarcoma, the microtubule inhibitor ibrine mesylate, and p53 MDM2 inhibitors such as RG7112 and RG7388 offers hope for reducing recurrence and limiting the need for repeated surgeries in patients with liposarcoma.^[23,24]^

This study is limited by its single-case design and short follow-up period, which may not fully capture long-term recurrence patterns. Additionally, advanced molecular profiling (e.g., whole-genome sequencing) was not performed, potentially missing actionable therapeutic targets.

## 4. Conclusion

RPLS is a rare malignant tumor with a high recurrence rate. Currently, surgical resection is the preferred treatment, with early detection and complete removal being critical to reducing recurrence. Comprehensive preoperative evaluation, multidisciplinary collaboration, precise surgical techniques, and standardized intraoperative and postoperative management are essential to improving resection rates and minimizing complications. This case highlights the importance of multidisciplinary collaboration in the diagnosis and treatment of retroperitoneal tumors. By combining imaging, histopathology, and molecular markers (such as MDM2), the diagnostic accuracy can be significantly improved. Additionally, raising public awareness of tumor prevention and treatment can further improve the prognosis of patients with RPLS.

## Author contributions

**Conceptualization:** Zicheng Bao, Nan Jia, Zhidong Zhang, Qun Zhao, Xuefeng Zhao.

**Formal analysis:** Zicheng Bao, Zhidong Zhang, Pingan Ding, Qun Zhao, Xuefeng Zhao.

**Funding acquisition:** Yong Li.

**Investigation:** Zicheng Bao, Nan Jia, Pingan Ding, Yong Li.

**Methodology:** Zicheng Bao, Pingan Ding, Yong Li.

**Project administration:** Nan Jia, Zhidong Zhang.

**Resources:** Zicheng Bao, Nan Jia.

**Supervision:** Nan Jia, Zhidong Zhang, Qun Zhao, Xuefeng Zhao.

**Validation:** Nan Jia.

**Writing – original draft:** Zicheng Bao.

**Writing – review & editing:** Zicheng Bao, Nan Jia, Zhidong Zhang, Pingan Ding, Yong Li.

## References

[1] Pasquali S Gronchi A. Neoadjuvant chemotherapy in soft tissue sarcomas: latest evidence and clinical implications. Ther Adv Med Oncol. 2017;9:415–29. 28607580 10.1177/1758834017705588 PMC5455882

[2] Makni A Triki A Fetirich F. Giant retroperitoneal liposarcoma. Report of 5 cases. Ann Ital Chir. 2012;83:161–6. 22462339

[3] Deacu M Bosoteanu M Enciu M. The predictive role of the histopathological scoring system in adipose tumors-lipoma, atypical lipomatous tumor, and liposarcoma. Diagnostics (Basel). 2023;13:3606. 38132190 10.3390/diagnostics13243606 PMC10742782

[4] Sbaraglia M Bellan E Tos APD. The 2020 WHO classification of soft tissue tumours: news and perspectives. Pathologica. 2021;113:70–84. 33179614 10.32074/1591-951X-213 PMC8167394

[5] Shibuya T Mori A Fushimi N. Pelvic retroperitoneal liposarcoma diagnosed by preoperative imaging studies. Intern Med. 2007;46:1263–4. 17675782 10.2169/internalmedicine.46.0242

[6] Ikeguchi M Urushibara S Shimoda R Saito H Wakatsuki T. Surgical treatment of retroperitoneal liposarcoma. Yonago Acta Med. 2014;57:129–32. 25901099 PMC4404522

[7] Wei X Qin Y Ouyang S Qian J Tu S Yao J. Challenging surgical treatment of giant retroperitoneal liposarcoma: a case report. Oncol Lett. 2022;24:314. 35949617 10.3892/ol.2022.13434 PMC9353788

[8] Ikoma N Torres KE Somaiah N. Accuracy of preoperative percutaneous biopsy for the diagnosis of retroperitoneal liposarcoma subtypes. Ann Surg Oncol. 2015;22:1068–72. 25354575 10.1245/s10434-014-4210-8 PMC4520392

[9] Serio G Tenchini P Nifosi F Iacono C. Surgical strategy in primary retroperitoneal tumours. Br J Surg. 1989;76:385–9. 2720349 10.1002/bjs.1800760423

[10] Linehan DC Lewis JJ Leung D Brennan MF. Influence of biologic factors and anatomic site in completely resected liposarcoma. J Clin Oncol. 2000;18:1637–43. 10764423 10.1200/JCO.2000.18.8.1637

[11] Lewis JJ Leung D Woodruff JM Brennan MF. Retroperitoneal soft-tissue sarcoma: analysis of 500 patients treated and followed at a single institution. Ann Surg. 1998;228:355–65. 9742918 10.1097/00000658-199809000-00008 PMC1191491

[12] Bonvalot S Miceli R Berselli M. Aggressive surgery in retroperitoneal soft tissue sarcoma carried out at high-volume centers is safe and is associated with improved local control. Ann Surg Oncol. 2010;17:1507–14. 20393803 10.1245/s10434-010-1057-5

[13] Strauss DC Hayes AJ Thway K Moskovic EC Fisher C Thomas JM. Surgical management of primary retroperitoneal sarcoma. Br J Surg. 2010;97:698–706. 20306527 10.1002/bjs.6994

[14] Pasquali S Gronchi A. Retroperitoneal liposarcoma: an aggressive strategy to maximize disease control. Int J Radiat Oncol Biol Phys. 2017;98:273–4. 10.1016/j.ijrobp.2017.01.230 28463147

[15] Bao Z Zhang Z Ding P Zhao Q Li Y. A case report of retroperitoneal liposarcoma. Medicine (Baltimore). 2024;103:e39633. 39287238 10.1097/MD.0000000000039633 PMC11404892

[16] Tan MCB Brennan MF Kuk D. Histology-based classification predicts pattern of recurrence and improves risk stratification in primary retroperitoneal sarcoma. Ann Surg. 2016;263:593–600. 25915910 10.1097/SLA.0000000000001149 PMC4619189

[17] Tuan J Vitolo V Vischioni B. Radiation therapy for retroperitoneal sarcoma. Radiol Med. 2014;119:790–802. 24638910 10.1007/s11547-013-0350-3

[18] Ecker BL Peters MG McMillan MT. Preoperative radiotherapy in the management of retroperitoneal liposarcoma. Br J Surg. 2016;103:1839–46. 27682864 10.1002/bjs.10305

[19] Rufo J Zhang P Zhong R Lee LP Huang TJ. A sound approach to advancing healthcare systems: the future of biomedical acoustics. Nat Commun. 2022;13:3459. 35710904 10.1038/s41467-022-31014-y PMC9200942

[20] Irshaid L Costigan DC Dong F Matulonis UA Nucci MR Kolin DL. Molecular landscape of mullerian clear cell carcinomas identifies the cancer genome atlas-like prognostic subgroups. Mod Pathol. 2023;36:100123. 36857998 10.1016/j.modpat.2023.100123

[21] Gardner RA Shah NN. CAR T-cells for cure in pediatric B-ALL. J Clin Oncol. 2023;41:1646–8. 36634289 10.1200/JCO.22.02345 PMC10043577

[22] Italiano A Bianchini L Gjernes E. Clinical and biological significance of CDK4 amplification in well-differentiated and dedifferentiated liposarcomas. Clin Cancer Res. 2009;15:5696–703. 19737942 10.1158/1078-0432.CCR-08-3185

[23] Blay JY Casali P Nieto A Tanović A Le Cesne A. Efficacy and safety of trabectedin as an early treatment for advanced or metastatic liposarcoma and leiomyosarcoma. Future Oncol. 2014;10:59–68. 23987833 10.2217/fon.13.163

[24] Ding Q Zhang Z Liu J-J. Discovery of RG7388, a potent and selective p53-MDM2 inhibitor in clinical development. J Med Chem. 2013;56:5979–83. 23808545 10.1021/jm400487c

